# Can technology life-cycles be indicated by diversity in patent classifications? The crucial role of variety

**DOI:** 10.1007/s11192-015-1639-x

**Published:** 2015-07-22

**Authors:** Loet Leydesdorff

**Affiliations:** Amsterdam School of Communication Research (ASCoR), University of Amsterdam, P.O. Box 15793, 1001 NG Amsterdam, The Netherlands

**Keywords:** Diversity, Patent classification, Technology life-cycle, Solar cells, PV

## Abstract

In a previous study of patent classifications in nine material technologies for photovoltaic cells, Leydesdorff et al. (Scientometrics 102(1):629–651, 2015) reported cyclical patterns in the longitudinal development of Rao–Stirling diversity. We suggested that these cyclical patterns can be used to indicate technological life-cycles. Upon decomposition, however, the cycles are exclusively due to increases and decreases in the variety of the classifications, and not to disparity or technological distance, measured as (1 − *cosine*). A single frequency component can accordingly be shown in the periodogram. Furthermore, the cyclical patterns are associated with the numbers of inventors in the respective technologies. Sometimes increased variety leads to a boost in the number of inventors, but in early phases—when the technology is still under construction—it can also be the other way round. Since the development of the cycles thus seems independent of technological distances among the patents, the visualization in terms of patent maps, can be considered as addressing an analytically different set of research questions.

## Introduction

In a previous study of nine material technologies for photovoltaic (PV) cells, Leydesdorff et al. (2015) found a cyclic pattern in Rao–Stirling diversity (Rao 1982; Stirling 2007) using the cosine for technological proximity (Jaffe 1986) and relative frequencies among patent classifications as variety. The cyclic patterns could be recognized by an expert in these technologies as a reflection of the development of technological life-cycles. In this communication, I decompose the cyclic pattern in the diversity in terms of variety and disparity, respectively. The patterns will also be related to other parameters such as the number of patents, inventors, and assignees. The conclusion is that the disparity does not play a role in generating the cycles, since they can also and even more precisely be indicated by a sole measure of the variety such as the Herfindahl–Hirschman or Simpson index. Spectral analysis confirms that only a single component (i.e., variety) drives the cyclic development. Furthermore, the cyclic pattern in the classifications is reflected in the number of inventors, but with a potential delay.

## Data

Recently, the US Patent and Trade Office (USPTO) and the European Patent Office (EPO) abandoned their respective classification systems of patents in favor of the Cooperative Patent Classifications (CPC). CPC builds on the International Patent Classifications (IPC) of the World Intellectual Property Organization (WIPO), by taking the first four digits from IPC version 8. However, CPC enhances the hierarchically organized IPC (v.8) by making it possible to add technology-specific tags such as for “nanotechnology” (Y01) or “technologies for mitigating climate change” (Y02) (Veefkind et al. 2012).

The new classifications thus provide us with the possibility to generate sets of patents representing advanced technologies with a level of precision perhaps comparable only to the medical subject headings (MeSH) of PubMed/Medline in the case of publications (Lundberg et al. 2006; Rotolo and Leydesdorff 2014). We downloaded from USPTO, all patents tagged with Y02E 10/54$ for nine material technologies in PV cells on August 20, 2013 (Y02E 10/541), and for the other eight technologies in October and November 2013. The nine technologies and the numbers of patents under study are shown in Table 1 (cf. Shibata et al. 2010).

**Table 1:** Nine material technologies for photovoltaic cells distinguished in the cooperative patent classifications (CPC)

CPC	Description	USPTO	Download date
Y02E 10/541	CuInSe2 material PV cells	419	August 20, 2013
Y02E 10/542	Dye sensitized solar cells	547	October 23, 2013
Y02E 10/543	Solar cells from Group II–VI materials	302	November 26, 2013
Y02E 10/544	Solar cells from Group III–V materials	882	November 26, 2013
Y02E 10/545	Microcrystalline silicon PV cells	148	November 26, 2013
Y02E 10/546	Polycrystalline silicon PV cells	269	November 26, 2013
Y02E 10/547	Monocrystalline silicon PV cells	1236	November 26, 2013
Y02E 10/548	Amorphous silicon PV cells	759	November 26, 2013
Y02E 10/549	Organic PV cells	1468	November 26, 2013

The data is indexed by professionals, so one would expect the distinctions between the nine technologies to be fine-grained and precise. Because some patents are tagged in more than a single category, the 6030 tags (in the third column of Table 1) are based on a smaller number of patents.

## Methods

Using VOSviewer (Van Eck and Waltman 2010) for the visualization, Leydesdorff et al. (2014) generated global maps on the basis of cosine-normalized vectors of the 124 IPC classes at the three-digit level and of the 630 IPC classes at the four-digit level. These maps can be used to project the IPCs in specific set(s) of patents under study in terms of both relative frequencies (size of the nodes) and distances on the map. The reader is referred to Leydesdorff et al. (2015) for more details and examples of the mapping and overlay techniques. In this study, we use the cosine values between the vectors of the 630 IPC classes at the four-digit level.^1^

Rao–Stirling diversity combines two of the three aspects of interdisciplinarity distinguished by Rafols and Meyer (2010): variety and disparity. [The third aspect, balance or coherence, was further developed by Rafols et al. (2012) for interdisciplinary units and by Leydesdorff and Rafols (2011) for developments at the field level.] Leydesdorff et al. (2013) added the value of Rao–Stirling diversity (Δ) routinely to the output as a measure of interdisciplinarity in the case of journal maps. What may be indicated by this same measure in the case of patent maps?

Rao–Stirling diversity is defined as follows (Rao 1982; Stirling 2007; cf. Zhang et al. 2014):

1 $$ \Delta = \sum\limits_{ij} {p_{i} p_{j} d_{ij} } $$

where *d*_*ij*_ is a disparity measure between two classes *i* and *j*—the categories are in this case IPC classes at the four-digit level—and *p*_*i*_ is the proportion of elements assigned to each class *i*. As the disparity measure, we use (1 − *cosine*) since the cosine values of the citation relations among the aggregated IPC were used for constructing the base map. Jaffe (1986, at p. 986) proposed taking the cosine between the vectors of classifications as a measure of “technological proximity.” In other words, we do not use the distances on the maps themselves, but the cosine values that were initially used for constructing the maps.

## Technology life-cycles

**Fig. 1:**
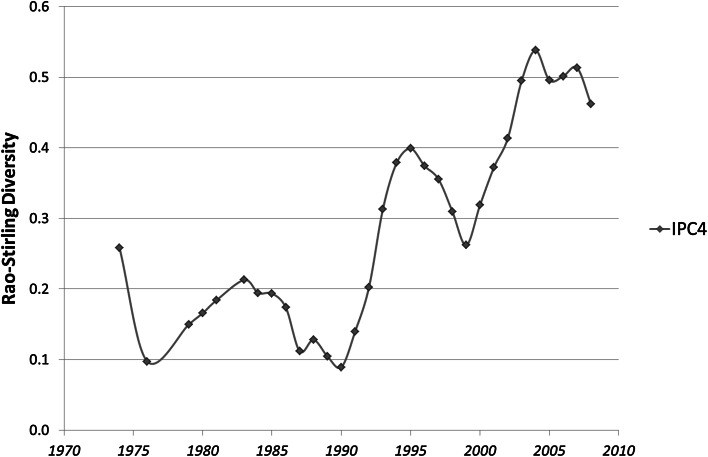
The development of Rao–Stirling diversity in IPC (three and four digits) among 419 USPTO-patents with CPC Y02E 10/541 (“CuInSe_2_ material PV cells”) during the period 1975–2012

Figure 1 shows the development of Rao–Stirling diversity using 419 USPTO-patents in the (first) CPC class under study (that is, Y02E 10/541) during the period 1975–2012. This figure suggests that the technology was developed in three cycles.

Two of the valleys, i.e., the period of decreasing diversity in the late 1980s and the most recent such period, correspond with breakthroughs in the efficiency of thin-film solar cells (Green et al. 2013). On the basis of analysis of co-invention addresses, expert interviews, and secondary literature, Leydesdorff et al. (2015, p. 640) specified these three cycles as follows (Shafarman and Stolt 2003):

1. an early cycle during the 1980s which is almost exclusively American; after initial development of the technology at Bell Laboratories in the 1970s, Boeing further developed the solar cells using these materials;
2. a second cycle during the 1990s that includes transatlantic collaboration and competition with Europe; the US, however, remains in the lead; and
3. a third and current cycle—the commercial phase—marked by the prevalence of American–Japanese collaboration and by collaboration *within* Europe.

Similar cycles were found using the other eight CPC classes under study.

Since Rao–Stirling diversity is composed of two components (variety and disparity), one can first ask which of the two components carries the cycles; or is it perhaps an interaction? Secondly, the cycles can perhaps be related to other attributes of the respective sets of patents, such as the numbers of patents, inventors, or assignees. Thirdly, one can correlate the longitudinal development of the nine technologies, and ask whether the developments have a single pattern in common; perhaps caused (for example) by changes in the policy of the patent office?

## The decomposition of Rao–Stirling diversity

**Fig. 2:**
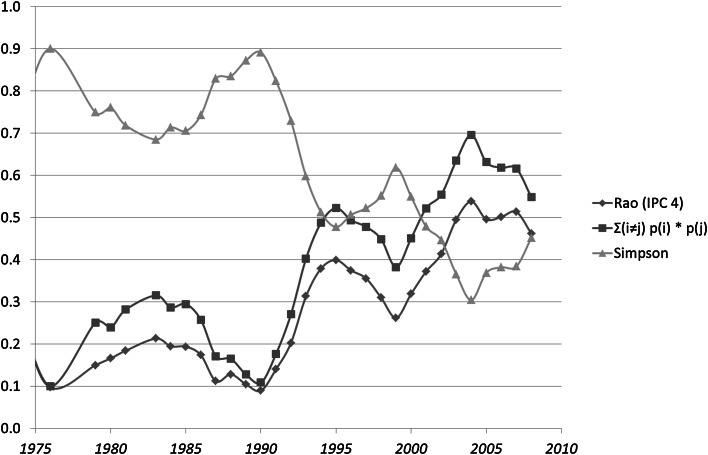
Rao–Stirling diversity, variety, and the Simpson Index for IPC four-digit classes in 419 USPTO-patents tagged CPC Y02E 10/541 (“CuInSe_2_ material PV cells”) during the period 1975–2012

If all disparity is equal to one (*d*_*ij*_ = 1), $\Delta = \sum\nolimits_{i \ne j} {p_{i} p_{j} }$. This is also called the Gini–Simpson index of diversity, and for analytical reasons, it is the complement to one of the Herfindahl–Hirsch index or equivalently the Simpson index (Stirling 2007).^2^ Figure 2 shows that the variety term under this assumption of all *d*_*ij*_ = 1 accounts for the cyclic development in Fig. 1.

Figure 2 shows that the cyclic pattern in Rao–Stirling diversity is caused by changes in the variety; the disparity is not needed for the explanation. Multiplication by a disparity measure (1 − *cosine*) attenuates the pattern exhibited using the Simpson (or Herfindahl) index. In sum, the latter indicator can be used for this analysis of diversity. Analysis of variety in the case of the other eight technologies led to similar results.

## Spectral analysis (Periodogram)

The question of whether one or two components are involved in the cycles can also be addressed using spectral analysis. In order to test this question, I performed spectral analysis of the curve in Fig. 1 using SPSS v.22. (Since spectral analysis requires an even number of observations, the first observation (1975) is not used.) Spectral analysis allows for testing an estimated spectrum in descriptive data without any a priori constraints (SPSS 1999, p. 205).

**Fig. 3:**
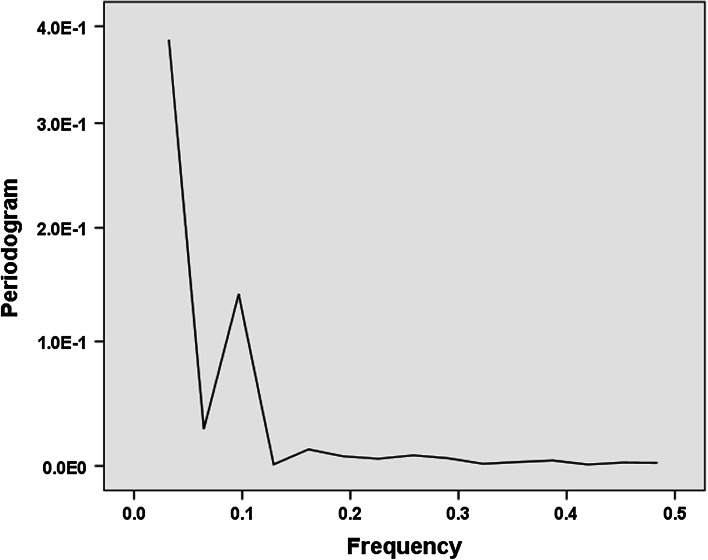
Periodogram of the development of Rao–Stirling diversity in IPC (four digits) among 419 USPTO-patents with CPC Y02E 10/541 (“CuInSe_2_ material PV cells”) during the period 1976–2012. (SPSS v.22)

**Fig. 4:**
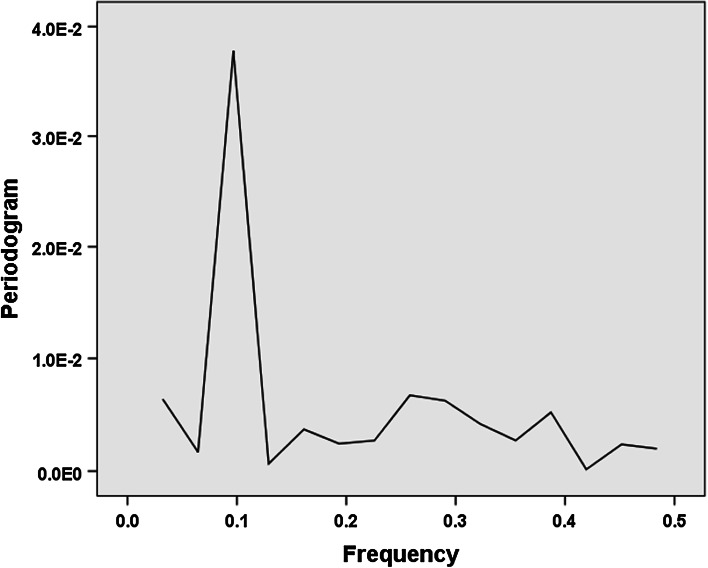
De-trended periodogram of the development of Rao–Stirling diversity in IPC (four digits) among 419 USPTO-patents with CPC Y02E 10/541 (“CuInSe_2_ material PV cells”) during the period 1976–2012. (SPSS v.22.)

The remaining 30 observations exhibit a single frequency at 0.1 (Fig. 3), indicating that three cycles are involved (3/30 = 0.1). The upshot on the left side of the figure indicates a linear trend—upward as visible in Fig. 1. De-trending the curve of Fig. 1 (using difference between consecutive years) provides Fig. 4.

This result confirms that a single component drives the cycles. This single component was identified above as variety.

## Other parameters

**Fig. 5:**
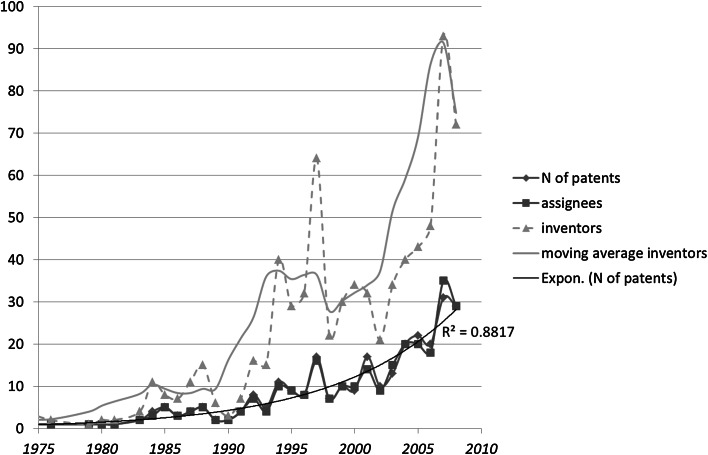
Numbers of patents, assignees, and inventors in 419 USPTO-patents tagged with CPC Y02E 10/541 (“CuInSe_2_ material PV cells”) during the period 1975–2012

Figure 5 shows that the numbers of patents and assignees in this set are highly correlated, and both show exponential growth during the period under study. The number of inventors, however, varies more. Patents in this domain (and in the others) tend to be assigned to a single assignee, whereas the number of co-inventors is less restricted.

**Fig. 6:**
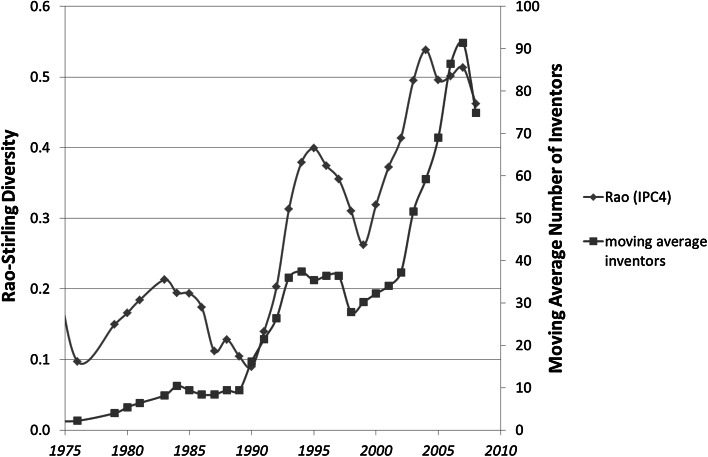
Rao–Stirling diversity and the number of inventors for 419 USPTO-patents tagged with CPC Y02E 10/541 (“CuInSe_2_ material PV cells”) during the period 1975–2012

The cyclic pattern in Fig. 1 can be retrieved by assuming similarly a 5-year moving average (MA) in the number of inventors (Fig. 6).

Figure 6 shows that the number of inventors lags behind the variety during the last cycle, but not during the valley around 1990. The relative lead of the variety when the volume has grown may indicate that the economic upswing in a technology attracts inventors more than that inventors are able to induce technological cycles in this more mature stage (Frenken and Leydesdorff 2000).

**Fig. 7:**
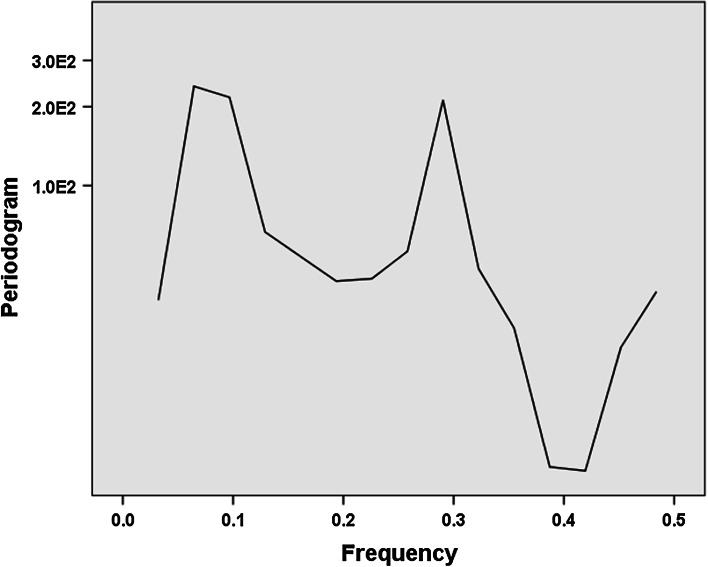
De-trended periodogram of the number of inventors for 419 USPTO-patents tagged with CPC Y02E 10/541 (“CuInSe_2_ material PV cells”) during the period 1975–2012

The de-trended periodogram of the number of inventors in Fig. 7 confirms that a second effect is to be distinguished in this case with a peak at 0.3, and thus indicating nine cycles (9/30 = 0.3). The cycles in the number of inventors can thus be distinguished from longer cycles in the technology.

**Fig. 8:**
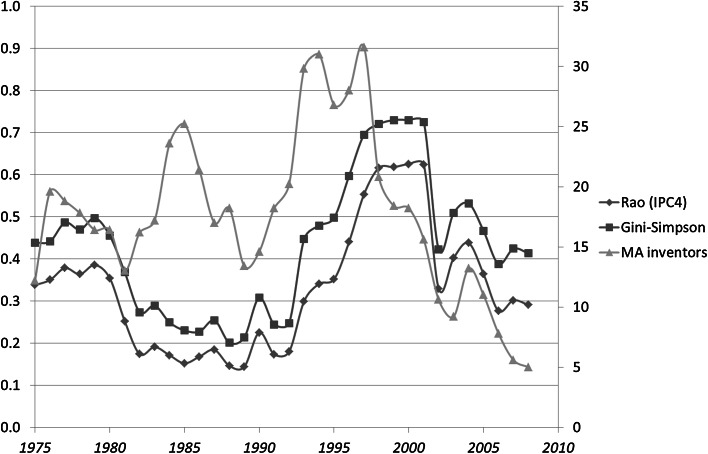
Rao–Stirling diversity, Gini–Simpson Index, and (5-year) moving averages of the number of inventors; 320 patents classified Y02E 10/543 (“Solar cells from Group II-VI materials”) in USPTO during the period 1975–2012

In the case of Y02E 10/543 (“Solar cells from Group II-VI materials”), for example, the numbers are smaller, and the moving average of the number of inventors leads the curve of the (Gini–Simpson) variety in this case (Fig. 8).

## Correlations

Spearman’s rank-order correlation coefficient (*ρ*) can be used to test the degree to which a monotonic relationship exists between two variables (Sheskin 2011, at p. 1366). Since the time-series increases monotonically in terms of sequential years, this measure allows us also to test for increasing or decreasing trends (Bornmann and Leydesdorff 2013).

Table 2 shows the Spearman correlation coefficients for the years since 1975 and the Gini–Simpson coefficients for the nine PV technologies. A number of these technologies (e.g., Y02E 10/541 and Y02E 10/548) show significantly (*p* < 0.01) increasing diversity over time. Y02E 10/544 and Y02E 10/546), however, are negatively correlated among them. Whereas the general pattern is one of increase, the indicator also shows differences among these technologies in terms of the Gini–Simpson index.

**Table 2:** Spearman rank-order correlations of time-series for Gini–Simpson coefficients, 1975–2012

	Year	c541	c542	c543	c544	c545	c546	c547	c548	c549
Year	1	.835**	.480**	0.33	−0.04	−0.43	0.17	.403*	.539**	0.19
c541	.835**	1	.410*	.539**	−0.02	−0.32	.531**	.766**	.625**	.532**
c542	.480**	.410*	1	.433*	.408*	0.25	−0.31	0.07	.653**	0.17
c543	0.33	.539**	.433*	1	.399*	0.21	0.21	.721**	.617**	0.26
c544	−0.04	−0.02	.408*	.399*	1	0.18	−.518**	0.17	0.32	0.19
c545	−0.43	−0.32	0.25	0.21	0.18	1	−0.04	−0.10	−0.14	−0.11
c546	0.17	.531**	−0.31	0.21	−.518**	−0.04	1	.549**	0.01	.554**
c547	.403*	.766**	0.07	.721**	0.17	−0.10	.549**	1	.488**	0.31
c548	.539**	.625**	.653**	.617**	0.32	−0.14	0.01	.488**	1	0.16
c549	0.19	.532**	0.17	0.26	0.19	−0.11	.554**	0.31	0.16	1

** Correlation is significant at the .01 level (2-tailed)

* Correlation is significant at the .05 level (2-tailed)

## Conclusion

The cyclical patterns in the Rao–Stirling diversity of nine technologically specific sets of patents were exclusively due to increases and decreases in the variety, and not in the disparity. The variety can, for example, be measured using the Simpson or Herfindahl index. The number of inventors is related to the development of the variety, but possibly with a temporal lag. In early phases of the technology, the development of the variety can be expected to lag, but in later stages the numbers of inventors tend to follow the development of variety in the patent classifications. The nine technologies under study, however, exhibit different patterns: when the technology is under construction the inventors tend to generate the variety, whereas in later stages the number of inventors tends to follow the development of the variety. Accordingly, the curve for the (moving average of the) number of inventors show three times as many cycles (in the periodogram) as the technologies (operationalized as patents). In other words, the technology cycles are relatively long (e.g., 10 years).

Whereas inventors follow or participate in constructing a research front, assignees can be considered primarily as economic agents who follow another (economic) logic than the technology cycles. Note that these conclusions are based on a specific set of technologies. Further research should show if variety can be used as a measure of technological development more generally. Our results suggest that the invention process has a dynamic of itself that is longer-termed than the cycling in the average number of inventors (Ivanova and Leydesdorff 2015). The inventors can then be considered as reflexively participating in retaining wealth from technological developments.

## Footnotes

1 The file with the 630 * 630 cosine values can be retrieved at http://www.leydesdorff.net/ipcmaps/cos_ipc4.dbf.

2 $\sum\nolimits_{ij} {p_{i} p_{j} } = 1$ when taken over all *i* and *j.* The Simpson index is equal to $\sum\nolimits_{i} {(p_{i} )^{2} }$, and the Gini–Simpson to $\left[ {1 - \sum\nolimits_{i} {(p_{i} )^{2} } } \right].$ Furthermore (Zhou et al. 2012, pp. 804f.):

$$ \begin{aligned} \sum\nolimits_{ij} {p_{i} p_{j} } & = \sum\nolimits_{i} {p_{i} p_{i} } + \sum\nolimits_{i \ne j} {p_{i} p_{j} } \\ 1 & = \sum\nolimits_{i} {p_{i} p_{i} } + \sum\nolimits_{i \ne j} {p_{i} p_{j} } \\ \sum\nolimits_{i} {p_{i} p_{i} } & = 1 - \sum\nolimits_{i \ne j} {p_{i} p_{j} } \\ \end{aligned} $$

Or, in other words: Simpson = 1 − variety. Note that for *i* = *j*—that is the diagonal—cosine(*i,i*) = 1, and the disparity (1 − cos) = 0. Therefore, this term does not contribute to the Rao–Stirling diversity in our case, and variety is equal to $\sum\nolimits_{i \ne j} {p_{i} p_{j} }$.

## References

[CR1] Bornmann L, Leydesdorff L (2013). Macro-indicators of citation impacts of six prolific countries: InCites data and the statistical significance of trends. PLoS ONE.

[CR2] Frenken K, Leydesdorff L (2000). Scaling trajectories in civil aircraft (1913–1997). Research Policy.

[CR23] Green, M. A., Emery, K., Hishikawa, Y., Warta, W., & Dunlop, E. D. (2013). Solar cell efficiency tables (version 41). *Progress in Photovoltaics: Research and Applications*, *21*(1), 1–11. doi:10.1002/pip.2352

[CR3] Ivanova IA, Leydesdorff L (2015). Knowledge-generating efficiency in innovation systems: The relation between structural and temporal effects. Technological Forecasting and Social Change.

[CR4] Jaffe AB (1986). Technological opportunity and spillovers of *R&D*: Evidence from firm’s patents, profits, and market value. American Economic Review.

[CR5] Leydesdorff L, Alkemade F, Heimeriks G, Hoekstra R (2015). Patents as instruments for exploring innovation dynamics: Geographic and technological perspectives on “photovoltaic cells”. Scientometrics.

[CR6] Leydesdorff L, Kushnir D, Rafols I (2014). Interactive overlay maps for US Patent (USPTO) data based on international patent classifications (IPC). Scientometrics.

[CR7] Leydesdorff L, Rafols I (2011). How do emerging technologies conquer the world? An exploration of patterns of diffusion and network formation. Journal of the American Society for Information Science and Technology.

[CR8] Leydesdorff L, Rafols I, Chen C (2013). Interactive overlays of journals and the measurement of interdisciplinarity on the basis of aggregated journal–journal citations. Journal of the American Society for Information Science and Technology.

[CR9] Lundberg J, Fransson A, Brommels M, Skår J, Lundkvist I (2006). Is it better or just the same? Article identification strategies impact bibliometric assessments. Scientometrics.

[CR10] Rafols I, Leydesdorff L, O’Hare A, Nightingale P, Stirling A (2012). How journal rankings can suppress interdisciplinary research: A comparison between innovation studies and business & management. Research Policy.

[CR11] Rafols I, Meyer M (2010). Diversity and network coherence as indicators of interdisciplinarity: Case studies in bionanoscience. Scientometrics.

[CR12] Rao CR (1982). Diversity: Its measurement, decomposition, apportionment and analysis. Sankhy: The Indian Journal of Statistics, Series A.

[CR13] Rotolo, D., & Leydesdorff, L. (2014, early view). Matching MEDLINE/PubMed data with web of science (WoS): A routine in R language. *Journal of the Association for Information Science and Technology.*. doi:10.1002/asi.23385. (forthcoming).

[CR14] Shafarman WN, Stolt L, Luque A, Hegedus S (2003). Cu (InGa) Se2 solar cells. Handbook of photovoltaic science and engineering.

[CR15] Sheskin DJ (2011). Handbook of parametric and nonparametric statistical procedures.

[CR16] Shibata N, Kajikawa Y, Sakata I (2010). Extracting the commercialization gap between science and technology—case study of a solar cell. Technological Forecasting and Social Change.

[CR17] SPSS (1999). SPSS Trends™ 10.

[CR18] Stirling A (2007). A general framework for analysing diversity in science, technology and society. Journal of the Royal Society, Interface.

[CR19] Van Eck NJ, Waltman L (2010). Software survey: VOSviewer, a computer program for bibliometric mapping. Scientometrics.

[CR20] Veefkind V, Hurtado-Albir J, Angelucci S, Karachalios K, Thumm N (2012). A new EPO classification scheme for climate change mitigation technologies. World Patent Information.

[CR21] Zhang, L., Rousseau, R., & Glänzel, W. (2014). Diversity of references as an indicator for interdisciplinarity of journals: Taking similarity between subject fields into account. *Journal of the Association for Information Science and Technology*. doi:10.1002/asi.23487.

[CR22] Zhou Q, Rousseau R, Yang L, Yue T, Yang G (2012). A general framework for describing diversity within systems and similarity between systems with applications in informetrics. Scientometrics.

